# The Accuracy and Bias of Single-Step Genomic Prediction for Populations Under Selection

**DOI:** 10.1534/g3.117.043596

**Published:** 2017-06-22

**Authors:** Wan-Ling Hsu, Dorian J. Garrick, Rohan L. Fernando

**Affiliations:** * Department of Animal Science, Iowa State University, Ames, Iowa 50011; † Institute of Veterinary, Animal and Biomedical Sciences, Massey University, Palmerston North 4442, New Zealand

**Keywords:** centering genotype covariates, estimated breeding value, genomic prediction, selection, single-step, GenPred, Shared Data Resources, Genomic Selection

## Abstract

In single-step analyses, missing genotypes are explicitly or implicitly imputed, and this requires centering the observed genotypes using the means of the unselected founders. If genotypes are only available for selected individuals, centering on the unselected founder mean is not straightforward. Here, computer simulation is used to study an alternative analysis that does not require centering genotypes but fits the mean $\mu_{g}$ of unselected individuals as a fixed effect. Starting with observed diplotypes from 721 cattle, a five-generation population was simulated with sire selection to produce 40,000 individuals with phenotypes, of which the 1000 sires had genotypes. The next generation of 8000 genotyped individuals was used for validation. Evaluations were undertaken with (J) or without (N) $\mu_{g}$ when marker covariates were not centered; and with (JC) or without (C) $\mu_{g}$ when all observed and imputed marker covariates were centered. Centering did not influence accuracy of genomic prediction, but fitting $\mu_{g}$ did. Accuracies were improved when the panel comprised only quantitative trait loci (QTL); models JC and J had accuracies of 99.4%, whereas models C and N had accuracies of 90.2%. When only markers were in the panel, the 4 models had accuracies of 80.4%. In panels that included QTL, fitting $\mu_{g}$ in the model improved accuracy, but had little impact when the panel contained only markers. In populations undergoing selection, fitting $\mu_{g}$ in the model is recommended to avoid bias and reduction in prediction accuracy due to selection.

In pedigree-based analyses, the expected value of breeding values is zero. In order to achieve similar properties in whole-genome analyses, marker genotype covariates are often transformed. When all individuals are genotyped, it has been shown that inference on genotype effects does not depend on how the covariates are transformed (Strandén and Christensen 2011). However, when data includes genotyped and nongenotyped individuals, inference on marker effects from single-step analyses may depend on how the covariates are transformed (Fernando *et al.* 2014). In single-step analyses using marker effects models, the breeding values of nongenotyped individuals are partitioned into components representing the prediction of nongenotyped individuals conditional on their genotyped relatives and an independent deviation (Fernando *et al.* 2014). The prediction of nongenotyped individuals conditional on their genotyped relatives is done based on best linear prediction, which requires the first moments to be known without error. This is straightforward if the mean of the genomic breeding value is zero in the absence of selection. Centering the observed genotype covariates using what their means would be in the absence of selection would result in genomic breeding values with null means. However, such genotype covariate means are typically unavailable. Fernando *et al.* (2014) proposed a solution for the marker effects model that involves fitting an additional fixed covariate that estimates the mean $\mu_{g}$ of the linear component of the genotypic value, which is denoted by $a_{i}$ in Equation 1 below, in a population where selection is absent. Using this approach, even when there is selection, the selection process can be ignored, because the analysis is conditional on the data used for selection (Goffinet 1983; Gianola and Fernando 1986; Im *et al.* 1989; Sorensen *et al.* 2001). In Markov chain Monte Carlo (MCMC) analyses, centering results in better mixing (Strandén and Christensen 2011), reducing the number of iterations required to obtain converged genomic predictions. In practice, centering the entire matrix of genotype covariates, including the observed and imputed genotypes, using their sample means can be done in addition to the Fernando *et al.* (2014) approach of fitting $\mu_{g}.$ This type of centering of the entire genotype matrix does not affect inference on marker effects.

The same issue with centering of the observed genotype covariates that we described above for the marker effects model is also implicit for the single-step breeding value model (single-step GBLUP), and a similar solution was proposed by Vitezica *et al.* (2011). In their proposed solution, the observed genotype covariates are centered using their means; in addition, the genomic covariance matrix is corrected for the change in the mean breeding value of the genotyped individuals (Vitezica *et al.* 2011). It was shown in that paper that this is equivalent to fitting the change in breeding value due to selection as a random effect.

Here, we use simulated data to compare the accuracy and bias in genomic prediction applied to populations under selection with and without centering the entire matrix of genotype covariates, and with and without fitting $\mu_{g}$ as a fixed effect. Further, we show that when the observed genotype covariates are centered using means calculated from selected individuals rather than means from all individuals, the meaning of $\mu_{g}$ changes from the mean of unselected individuals to become the mean breeding value in selected individuals, as claimed by Vitezica *et al.* (2011).

## Methods

### Theory

To simplify the presentation of the genetic model without loss of generality, we will assume that the unconditional expectation of the phenotypic value for all individuals is the same. Let $m_{i}^{\prime }$ denote the row vector of genotypes for individual *i*, which is often coded as −1,0,1. Then, under additive gene action, the genotypic value, $g_{i},$ which is the expected phenotypic value of an individual with genotypes $m_{i}^{\prime }$, can be written as

$$\begin{array}{c} g_{i}=\beta +m_{i}^{\prime }\alpha ,\\ =\beta +a_{i} \end{array}$$ (1)

where *β* is the value of $g_{i}$ when $m_{i}^{\prime }=0^{\prime }$ and α is the vector of substitution effects. The scalar *β* and the vector α are constants, but $g_{i}$ will be a random variable because of randomness in $a_{i}=m_{i}^{\prime }\alpha ,$ owing to the randomness in the genotypes for a randomly sampled individual. Note that the expected value of the linear component $a_{i}$ of the genotypic value in Equation 1 is $\text{E}\left(a_{i}\right)=\text{E}\left(m_{i}^{\prime }\right)\alpha =k^{\prime }\alpha =\mu_{g},$ where $k^{\prime }=\text{E}\left(m_{i}^{\prime }\right),$ which may not be equal to zero. Thus, it is customary to write the model for the genotypic value, as can be derived from Equation 1, as follows:

$$\begin{array}{c} g_{i}=\left(\beta +\mu_{g}\right)+a_{i}-\mu_{g}\\ =\left(\beta +\mu_{g}\right)+u_{i}\\ =\left(\beta +\mu_{g}\right)+\left(m_{i}^{\prime }-k^{\prime }\right)\alpha , \end{array}$$ (2)

where $\left(\beta +\mu_{g}\right)$ is a constant, representing $\text{E}\left(g_{i}\right),$ and $u_{i}=\left(m_{i}^{\prime }-k^{\prime }\right)\alpha$ is a random variable that has null expectation, which is the breeding value predicted in a pedigree-based BLUP (PBLUP) evaluation. When genotypes are observed and used in a genomic analysis, they may be transformed or coded by subtracting their expectations, $k^{\prime },$ from the observed values, $m_{i}^{\prime }.$ In both Equations 1 and 2, $\alpha_{j}$ is the same substitution effect for locus *j*. The intercepts in these models, however, are different. In Equation 1, the intercept is *β*, and it is the value of $g_{i}$ when $m_{i}^{\prime }=0^{\prime }.$ In Equation 2, on the other hand, the intercept is $\left(\beta +\mu_{g}\right),$ and it is the value of $g_{i}$ when $m_{i}^{\prime }=k^{\prime }.$ More generally, $k^{\prime }$ is not known, so genotypes are coded by subtracting a different vector $v^{\prime }$ from the observed genotypes as $m_{i}^{\prime }-v^{\prime }.$ Still, $\alpha_{j}$ is the substitution effect for locus *j*, but the intercept will change to become $\left(\beta +v^{\prime }\alpha \right),$ which is the value of $g_{i}$ when $m_{i}^{\prime }=v^{\prime }.$ Thus, as more rigorously shown in (Strandén and Christensen 2011), inference about α does not depend on how the genotypes are coded. A simpler but rigorous proof is given in the Appendix of this paper.

In single-step analyses, where some individuals are not genotyped, the missing genotypes are imputed either implicitly (Legarra *et al.* 2009) or explicitly (Fernando *et al.* 2014) using best linear prediction. Let $M_{g}$ denote the matrix of genotypes for individuals that were genotyped. Then, the genotypes of the individuals with missing genotypes are imputed as

$$M_{n}=1k^{\prime }+A_{ng}A_{gg}^{-1}\left(M_{g}-1k^{\prime }\right),$$

where $A_{ng}$ is the matrix of pedigree-based additive relationships between the nongenotyped and genotyped individual, and $A_{gg}$ is the matrix of additive relationships among genotyped individuals. Now, the model for the genotypic values, when genotypes are coded as in Equation 2, becomes

$$\begin{array}{l} g_{n}=1\left(\beta +\mu_{g}\right)+A_{ng}A_{gg}^{-1}\left(M_{g}-1k^{\prime }\right)\alpha +\epsilon \\ g_{g}=1\left(\beta +\mu_{g}\right)+\left(M_{g}-1k^{\prime }\right)\alpha . \end{array}$$

where ϵ is that part of $g_{n}$ that cannot be imputed from knowledge of the breeding values of genotyped relatives. In practice, the true value of $k^{\prime }$ is not known, and data for its estimation may not be available. Rearranging these equations in terms of the uncentered $M_{g}$ rather than the centered matrix of genotype covariates $\left(M_{g}-1k^{\prime }\right),$ results in

$$\begin{array}{l} g_{n}=1\left(\beta +\mu_{g}\right)-A_{ng}A_{gg}^{-1}1k^{\prime }\alpha +A_{ng}A_{gg}^{-1}M_{g}\alpha +\epsilon \\ g_{g}=1\left(\beta +\mu_{g}\right)-1k^{\prime }\alpha +M_{g}\mathrm{\alpha ,} \end{array}$$

and substituting $\mu_{g}=k^{\prime }\alpha ,$ as previously defined, and letting ${\hat{M}}_{n}=A_{ng}A_{gg}^{-1}M_{g},$ results in

$$\begin{array}{l} g_{n}=1\left(\beta +\mu_{g}\right)-A_{ng}A_{gg}^{-1}1\mu_{g}+{\hat{M}}_{n}\alpha +\epsilon \\ g_{g}=1\left(\beta +\mu_{g}\right)-1\mu_{g}+M_{g}\mathrm{\alpha ,} \end{array}$$ (3)

which suggests that $\mu_{g}=k^{\prime }\alpha$ could be treated as an unknown constant and estimated as a fixed effect from the data (Fernando *et al.* 2014). The covariate vector for $\mu_{g}$ is denoted by $J_{n}=-A_{ng}A_{gg}^{-1}1$ for nongenotyped individuals, and by $J_{g}=-1$ for genotyped individuals. So, Equation 3 becomes

$$\begin{array}{l} g_{n}=1\left(\beta +\mu_{g}\right)+J_{n}\mu_{g}+{\hat{M}}_{n}\alpha +\epsilon \\ g_{g}=1\left(\beta +\mu_{g}\right)+J_{g}\mu_{g}+M_{g}\alpha , \end{array}$$

which can be combined as

$$g=1\left(\beta +\mu_{g}\right)+J\mu_{g}+M\alpha +\left[\begin{array}{c} I\\ 0 \end{array}\right]\epsilon ,$$ (4)

where $g=\left[\begin{array}{c} g_{n}\\ g_{g} \end{array}\right],$ $J=\left[\begin{array}{c} J_{n}\\ J_{g} \end{array}\right],$ $M=\left[\begin{array}{c} {\hat{M}}_{n}\\ M_{g} \end{array}\right],$ and the **0** matrix in the last term of Equation 4 is required because ϵ does not appear in the model for genotyped individuals.

When the vector α represents the substitution effects of a large number of loci containing positive and negative effects, $\mu_{g}=k^{\prime }\alpha$ will tend to have a value close to zero. Accordingly, we have simulated some scenarios with positive $\mu_{\alpha }=\text{E}\left(\alpha_{i}\right)$ so that the entire α vector is positive to exacerbate the impact of $\mu_{g}=k^{\prime }\alpha .$ Nevertheless, when marker rather than causal alleles are fitted in the model, the sign of the substitution effects depends on the phase relationship between marker and causal allele, which is equally likely to be positive or negative.

Even if $\mu_{\alpha }=\text{E}\left(\alpha_{i}\right)=0,$ in a population undergoing selection, it is expected that $\text{E}\left(a_{i}\right)=\text{E}\left(m_{i}^{\prime }\right)\alpha \neq 0$ in nonfounders. Suppose $v^{\prime }$ is the mean of the observed genotype covariates in such a population undergoing selection, and these means are used to center the matrix $M_{g}$ of observed genotypes. Then, the model for the genotypic values can be written in terms of the matrix $M_{g}^{\text{*}}=M_{g}-1v^{\prime }$ of centered covariates as

$$\begin{array}{l} g_{n}=1\left(\beta^{\text{*}}+\mu_{g}^{\text{*}}\right)-A_{ng}A_{gg}^{-1}1\mu_{g}^{\text{*}}+A_{ng}A_{gg}^{-1}M_{g}^{\text{*}}\alpha +\epsilon \\ g_{g}=1\left(\beta^{\text{*}}+\mu_{g}^{\text{*}}\right)-1\mu_{g}^{\text{*}}+M_{g}^{\text{*}}\alpha , \end{array}$$ (5)

and using J for the covariate corresponding to $\mu_{g\text{,}}^{\text{*}}$ Equation 5 can be written as

$$\begin{array}{c} g_{n}=1\left(\beta^{\text{*}}+\mu_{g}^{\text{*}}\right)+J_{n}\mu_{g}^{\text{*}}+A_{ng}A_{gg}^{-1}\left(M_{g}-1v^{\prime }\right)\alpha +\epsilon \\ =1\left(\beta^{\text{*}}+\mu_{g}^{\text{*}}\right)+J_{n}\mu_{g}^{\text{*}}+{\hat{M}}_{n}\alpha +J_{n}v^{\prime }\alpha +\epsilon \\ g_{g}=1\left(\beta^{\text{*}}+\mu_{g}^{\text{*}}\right)+J_{g}\mu_{g}^{\text{*}}+\left(M_{g}-1v^{\prime }\right)\alpha \\ =1\left(\beta^{\text{*}}+\mu_{g}^{\text{*}}\right)+J_{g}\mu_{g}^{\text{*}}+M_{g}\alpha +J_{g}v^{\prime }\mathrm{\alpha ,} \end{array}$$

which can be combined as

$$g=1\left(\beta^{\text{*}}+\mu_{g}^{\text{*}}\right)+J\left(\mu_{g}^{\text{*}}+v^{\prime }\alpha \right)+M\alpha +\left[\begin{array}{c} I\\ 0 \end{array}\right]\epsilon .$$ (6)

Note that the regression coefficients for J, $\mu_{g}$ in Equation 4 and $\mu_{g}^{\text{*}}+v^{\prime }\alpha$ in Equation 6, must be equal. This implies that $\mu_{g}^{\text{*}}=\mu_{g}-v^{\prime }\alpha .$ Similarly, the intercepts of these two models must be equal too, and this implies $\beta^{\text{*}}=\beta +v^{\prime }\alpha .$

### Simulations

Phenotypic and genotypic data were simulated using XSim (Cheng *et al.* 2015), based on haplotypes from 10 genomic regions of 721 US Hereford beef cattle that were genotyped with the Illumina 770K BovineHD BeadChip, and reported in terms of the number of copies of the A allele at each locus. The selected regions came from choosing every 10th single nucleotide polymorphism (SNP), starting at SNP 5001, to get a low-density panel of 200 SNPs from each of chromosomes 1–10 (BTA1–BTA10), after eliminating SNPs with MAF < 0.01. These 2000 SNPs represent 10 0.1 M chromosomes. Average linkage disequilibrium (LD) between adjacent SNPs was 0.300. 50 SNPs from each chromosome were randomly chosen to represent QTL. The QTL effects were sampled from a normal distribution with mean $\mu_{\alpha }$= 0.2 and multiplied by the number of copies of the A allele to produce the true breeding value (TBV). The TBVs were added to a normally distributed residual term scaled by the sample variance of the TBV to simulate a trait with a heritability of 0.5. The first 20 SNPs from each of the 10 chromosomes were also used to simulate a smaller panel, with 5 QTL and 15 markers per chromosome. The average LD between adjacent SNPs was 0.289. TBV were simulated in the same way as for the 2000 SNP scenario, then scaled to simulate traits with heritabilities ($h^{2}$) of 0.1, 0.3, or 0.5. An additional scenario with $\mu_{\alpha }$= 0 was used to simulate TBV for a trait with heritability 0.5.

Half the observed diplotypes from US Hereford cattle were assigned to represent males and the remainder to represent females. Those 360 males and 361 females were sampled in pairs, with replacement, to produce 4000 male and 4000 female offspring representing generation G4. There were no mutations. Four more nonoverlapping generations of random mating were carried out, with one male and one female offspring per dam mated to randomly chosen sires to produce the founder population (G0).

The G1 generation was produced by mass phenotypic selection of the top 200 G0 males, and this was repeated for five more generations. Each female was randomly mated twice to selected males to produce one offspring of each sex each generation. Across nonoverlapping generations G0–G5, a total of 48,000 individuals with phenotypes, genotypes, and TBVs were simulated for each scenario.

The training data included phenotypes from all individuals in G0–G4 (*n* = 40,000), and genotypes from all 1000 sires and all 8000 G5 animals. Fixed loci, if any, were filtered from the panel before genomic prediction analyses. The genetic and residual variances used in genomic prediction were the sample variance of the TBV in G0 and the corresponding residual variance used to define the desired heritability in the founder population.

### Models

Five statistical models were compared for differences in accuracy and bias of prediction. These included models with or without $\mu_{g}$, and with or without centering of observed and imputed marker covariates, and a model that used pedigree relationships but not marker covariates.

#### Mixed linear model:

Accuracy of PBLUP was quantified using the correlation of TBV and estimated breeding values (EBV), where TBV was as simulated and EBV were obtained by fitting the mixed linear model (Henderson 1973, 1984):

y=1μ+Zu+e,

where y is a vector of phenotypic observations, **1** is a vector of 1s, $\mu =\beta +\mu_{g}$ is a general mean, u is a vector of random additive genetic effects, e is a vector of random residual effects, and Z is a known incidence matrix relating observations to u. In this model, E(u)=0, E(e)=0, so that E(y)=1μ. Further, $\mathrm{Var}\left(u\right)=A\sigma_{g}^{2},$ with A being the numerator relationship matrix, and $\mathrm{Var}\left(e\right)=I\sigma_{e}^{2},$ so that $\mathrm{Var}\left(y\right)=ZAZ^{\prime }\sigma_{g}^{2}+I\sigma_{e}^{2}.$

#### Single-step Bayesian regression model:

All genomic EBVs (GEBV) were obtained by single-step Bayesian regression (Fernando *et al.* 2014), using BayesC priors for marker effects with *π* = 0, which gives predictions that are identical to those from single-step GBLUP. The model was implemented in Julia (http://julialang.org) based on the SSBR package (http://QTL.rocks) to construct an MCMC chain of 50,000 samples. Individuals were separated into two groups designated with subscripts *g* or *n* according to whether or not simulated genotypes were assumed to be observed or missing. The single-step Bayesian regression model including a covariate **J** for $\mu_{g}$ (model J) was:

$$\begin{array}{c} \left[\begin{array}{c} y_{n}\\ y_{g} \end{array}\right]=1\mu +\left[\begin{array}{c} Z_{n}J_{n}\\ Z_{g}J_{g} \end{array}\right]\mu_{g}+\left[\begin{array}{c} Z_{n}{\hat{M}}_{n}\\ Z_{g}M_{g} \end{array}\right]\alpha \\ +\left[\begin{array}{c} Z_{n}\\ 0 \end{array}\right]\epsilon +\left[\begin{array}{c} e_{n}\\ e_{g} \end{array}\right], \end{array}$$

where $y_{n}$ and $y_{g}$ are vectors of phenotypes for nongenotyped and genotyped individuals, **1** is a vector of 1s, *μ* is a general mean, $\mu_{g}$ is the expected value of the linear component $a_{i}$ of the genotypic value if selection was absent, α is a vector of random substitution effects of markers, ϵ is a vector of imputation residuals, $Z_{n}$ and $Z_{g}$ are incidence matrices relating the breeding values of nongenotyped and genotyped individuals to their phenotypes, $J_{g},$ which is defined for genotyped individuals, is a vector of −1s, $J_{n},$ which is defined for nongenotyped individuals, is a vector computed as $A_{ng}A_{gg}^{-1}J_{g},$ ${\hat{M}}_{n}$ is the matrix of imputed marker covariates, $M_{g}$ is the matrix of observed marker covariates, and $e_{n}$ and $e_{g}$ are vectors of random residual effects for nongenotyped and genotyped individuals. This model can be represented as

$$y=1\mu +ZJ\mu_{g}+ZM\alpha +U\epsilon +e,$$ (7)

where $y=\left[\begin{array}{c} y_{n}\\ y_{g} \end{array}\right],$ $Z=\left[\begin{array}{cc} Z_{n} & 0\\ 0 & Z_{g} \end{array}\right],$ $J=\left[\begin{array}{c} J_{n}\\ J_{g} \end{array}\right],$ $M=\left[\begin{array}{c} {\hat{M}}_{n}\\ M_{g} \end{array}\right],$ **U** = $\left[\begin{array}{c} Z_{n}\\ 0 \end{array}\right],$ and e is a vector of random residual effects.

This model was used for four variants of the single-step Bayesian regression analysis, depending on whether or not the covariate **J** corresponding to the mean $\mu_{g}$ was in the model, and whether or not the columns in the marker covariate matrix M were centered using means of the observed and imputed genotype covariates. The analyses with **J** or without **J** are denoted as J or N when covariates were not centered, and as JC or C when the entire matrix of imputed and observed genotype covariates were centered, respectively (Table 1).

**Table 1:** Four combinations of the single-step Bayesian regression analyses

Models	Marker Covariates	
	Centered*^a^*	Not Centered*^b^*
With J and **$\mu_{g}$**	JC	J
Without J	C	N

a Centered, *e.g.*, genotype values represented as −1, 0, 1 when the uncentered genotype covariate with values 0, 1, 2 has mean 1.

b Not centered, *e.g.*, genotype values represented as the number of copies of the A allele.

Accuracy of genomic prediction was quantified using the correlation of TBV and GEBV ($r_{g,\hat{g}}$), where GEBVs were obtained from each of the four analyses described above. Bias of genomic prediction was quantified using the deviation from unity of the coefficient of regression of TBV on GEBV ($b_{g,\hat{g}}$). In models JC and J, GEBVs are obtained using Equation 24 in (Fernando *et al.* 2014):

$$\hat{g}=J{\hat{\mu }}_{g}+M\hat{\alpha }+U\hat{\epsilon },$$

where $\hat{g}$ is the GEBV, ${\hat{\mu }}_{g}$ is the best linear unbiased estimate of the mean of breeding values, $\hat{\alpha }$ is the BLUP of the vector of random substitution effects of all markers, and $\hat{\epsilon }$ is the BLUP of the imputation residual.

In model J, the matrix $M_{g}$ contains the uncentered number of copies of the A allele at each locus, and the uncentered version is used to impute ${\hat{M}}_{n}.$ In model JC, the entire matrix of imputed, ${\hat{M}}_{n},$ and observed, $M_{g},$ genotype covariates is centered. In models C and N, the GEBVs are computed in a corresponding manner, except that the covariate ***J*** and its coefficient $\mu_{g}$ are not included in the model.

The four analyses J, N, JC, and C were all applied to three different genotype panels, comprising the causal QTL plus markers, just the causal QTL, or just the markers. All 12 combinations of four analyses and three genotype panels were applied to data simulated with 200 loci comprising 50 QTL whose effects were sampled from a normal distribution with $\mu_{\alpha }=0.2$ to construct phenotypes with $h^{2}=0.5.$ The four analyses JC, J, C, and N were repeated using only genotype panels comprising QTL plus markers for three other scenarios: 200 loci, $h^{2}=0.1,$ $\mu_{\alpha }=0.2;$ 200 loci, $h^{2}=0.3,$ $\mu_{\alpha }=0.2;$ and 200 loci, $h^{2}=0.5,$ $\mu_{\alpha }=0.2.$

Fernando *et al.* (2014) had observed that including $J_{g}$ in the model was necessary only when $\mu_{\alpha }\neq 0$ and the number of observations exceeds the number of markers. So, to examine the impact of $\mu_{\alpha },$ the scenario with 200 loci and $h^{2}=0.5$ was repeated with $\mu_{\alpha }=0.0,$ and to examine the impact of the number of loci, the scenario with $h^{2}=0.5$ and $\mu_{\alpha }=0.2$ was repeated with 2000 loci.

Every scenario was replicated 10 times with each replicate having been constructed starting from the sampling of G5 which represented simulated offspring from the haplotypes of real animals. Every phenotypic dataset was also fitted using the PBLUP model. All reported correlations and regression coefficients are the means of 10 replicates. These are presented along with the SEs of those means.

In single-step GBLUP (Legarra *et al.* 2009; Aguilar *et al.* 2010; Christensen and Lund 2010), the missing genotypes are not explicitly imputed, and only the observed genotype covariates are centered using their means. We argued earlier that when the number of loci is large, $\mu_{g}=k^{\prime }\alpha$ will be close to zero, especially when the panel consists of only marker loci. However, in a population undergoing selection, $\mu_{g}^{\text{*}}=\mu_{g}-v^{\prime }\alpha ,$ which is the mean breeding value of genotyped individuals, is not expected to be zero even when the panel only includes marker loci and $\mu_{\alpha }=0.$ So, in addition to the above, single-step Bayesian regression analyses, using the model given in Equation 7 with and without **J** (models JC* and C*), were applied to a marker panel with 200 loci, $h^{2}=0.5,$ and $\mu_{\alpha }=0,$ when the matrix of observed genotype covariates were centered using their means as: $M_{g}^{\text{*}}=M_{g}-1v^{\prime },$ where $v^{\prime }=1/n_{g}1^{\prime }M_{g},$ and $n_{g}$ is the number of individuals with genotypes. Recall that even when the means of the observed genotypes are used for centering, the model for the genotypic values can be written in terms of the matrix $M_{g}$ of uncentered covariates as shown by Equation 6. We will compare the estimates of $\mu_{g}^{\text{*}}$ in this model with those of $\mu_{g}$ from Equation 4 where the means of observed genotype covariates are not used for centering.

### Data availability

The genotypes representing G0 from each replicate and scenario are available at: https://figshare.com/s/d7798b811a9a6a4172fc. These genotypes and the methodology described previously are sufficient to reproduce the simulations used in this study.

## Results and Discussion

### Effect of fitting a genotypic mean and centering of observed and imputed marker covariates

#### Accuracy:

The accuracies of genomic prediction as assessed by validation in G5 after training using G0–G4 for a trait with 50 QTL whose effects were sampled from a normal distribution with $\mu_{\alpha }$= 0.2 and $h^{2}$= 0.5 are in Table 2. The accuracy of PBLUP in predicting breeding values for individuals without phenotypes was 41.5%, accounting for <20% of genetic variance. All analyses using genotypes resulted in more accurate predictions than using pedigree alone. Centering the entire matrix of imputed and observed genotype covariates had no effect on the accuracy of the genomic analyses regardless of the nature of the marker panel. This is because, as shown in the Appendix, this type of centering is equivalent to adding and subtracting $1{\bar{m}}^{\prime }\alpha$ from the model equation, and this has no effect on the mixed model solutions for α.

**Table 2:** Correlations (%, $\pm SE_{s}$) between TBV and (G)EBV for alternative analyses

Genotype Data*^a^*	Analyses				
	JC*^b^*	J*^c^*	C	N	PBLUP
50 QTL + 150 markers	97.59 ± 0.00	97.63 ± 0.00	96.32 ± 0.00	96.31 ± 0.00	—
50 QTL only	99.44 ± 0.00	99.45 ± 0.00	90.20 ± 0.05	90.18 ± 0.05	—
150 markers only	80.47 ± 0.03	80.47 ± 0.03	80.44 ± 0.03	80.44 ± 0.03	—
No genotypes	—	—	—	—	41.56 ± 0.01

a Average correlation between true breeding value (TBV) and (genomic) estimated breeding values from 10 replications validated in Generation 5, comprising 8,000 individuals with genotypes but no phenotypes. The true QTL effects were sampled from a Normal distribution with mean $\mu_{\alpha }$= 0.2 and scaled to simulate a trait with a heritability 0.5.

b J: includes a covariate for *μ_g_* in the model, C: entire matrix of imputed and observed genotype covariates centered, JC: both J and C, N: neither J or C, and PBLUP: pedigree-based BLUP.

c The analyses were based on fitting covariates for only 50 QTL, only 150 markers, or both 50 QTL and 150 markers.

In this study, selection resulted in the successive advances in mean TBV from G0 to G5 being 10.14, 10.63, 11.18, 11.68, 12.15, and 12.57. The mean genotypic value was not zero in G0 because QTL genotypes were not centered and the mean QTL effect was $\mu_{\alpha }$ = 0.2. Since the QTL effects do not change with selection, the advance in TBV reflects changes in the frequencies of the favorable alleles of the 50 QTL. So centering using the allele frequency means of the genotyped sires in G1–G4 and all individuals in G5 does not closely approximate the centering that would have occurred if the allele frequency means had been obtained from the unselected population. By contrast, fitting $\mu_{g}$ in the model estimates the relevant mean from the data.

In panels that included causal variants (QTL), fitting $\mu_{g}$in the model substantially improved the accuracy to being near perfect. This is not surprising, given that there were only 50 QTL, the heritability was 0.5, and there were 40,000 phenotyped ancestors, including 200 genotyped sires per generation in the training. However, in the panel that contained only markers with no causal variants, fitting $\mu_{g}$in the model had little impact. This is because in the population that was simulated here the founders were not selected, and thus $\mu_{g}$was close to zero for the panel with only markers. This would not be the case in a population where even the founders are descendants of selected parents. Thus, for traits that have been subject to selection, fitting J in the model is expected to improve accuracy.

Using one replicate as an example, for the panel including both QTL and markers, the estimate of *μ* was ∼9.90 for the analyses using J and N. The estimate of *μ_g_* was 4.98 for the analysis using J. For the genotyped individuals, the covariate values in $J_{g}$ are all −1, so $1\hat{\mu }$ + $J_{g}{\hat{\mu }}_{g}$ is a vector of values equal to 9.90−4.98 = 4.92. For nongenotyped individuals, the covariate values in $J_{n}$ can vary widely, but many are close to −1 while others are close to 0. This means that $1\hat{\mu }+J_{n}{\hat{\mu }}_{g}$ will include values that range from 9.90 to 4.92, accounting for variation in accuracy of imputation. When $\mu_{g}$ is not included in the model, these effects are ignored, which can reduce the accuracy of predicting nongenotyped individuals. Failing to account for these effects will propagate errors in $\hat{\epsilon }$ and $\hat{\alpha },$ the latter impacting the accuracy of predicting genotyped individuals. Collectively, these errors reduced accuracy from 97 to 96% for the panel including QTL and markers, and from 99 to 90% for the panel including only QTL. However, when the panel comprised only markers, the estimates $\hat{\alpha }$ will include both positive and negative values because the phase of markers and QTL are equally likely to take either sign, in which case ${\hat{\mu }}_{g}$ will be close to zero as confirmed in the above mentioned replicate where the estimate was −1.18.

Here, results from the analysis with only QTL on the panel are used to show that $\mu_{g}$ is the mean of $a_{i}$ in the founder population and not the mean of the breeding value $u_{i}$ of the genotyped individuals. Recall that the QTL model was used with an intercept of β=0.0 to simulate the data. Thus, when only QTL are on the panel, the true value of *β* is zero. In analysis J because $\mu =\left(\beta +\mu_{g}\right),$ both $\hat{\mu }$ and ${\hat{\mu }}_{g}$ are estimates of $\mu_{g},$ and could be pooled, which for the replicate above would be (9.91+8.19)/2=9.05. In that replicate, the actual mean of $a_{i}$ in G0 was 10.16, which was estimated in the analysis to be 9.05. On the other hand, the mean of the breeding value $u_{i}$ in the 9000 genotyped individuals was 2.4, which is clearly not near the pooled estimate of 9.05 for $\mu_{g}.$ These genotyped individuals included 1000 selected sires, of which 200 were genotyped in each generation from G0 to G4, and 8000 offspring from G5. The mean values of $u_{i}$ for the selected sires were 1.00, 1.59, 2.08, 2.50, and 2.88, respectively, for G0 through G4, and 2.45 for the offspring in G5. It is apparent that the $\mu_{g}$ parameter corresponding to the covariate J is the mean of $a_{i}$ in the founder population and not the mean breeding value $u_{i}$ in the selected individuals. In analysis JC with the covariates centered, the intercept *β* is the value of $g_{i}$ when $\left({m^{\prime }}_{i}-{v^{\prime }}_{i}\right)\alpha =0,$ which is the case when $m_{i}^{\prime }=v_{i}^{\prime }.$ The estimate $\hat{\mu }$ was 16.11 in this analysis, but ${\hat{\mu }}_{g}$ remained about the same value, namely 7.80. This shows that ${\hat{\mu }}_{g}$ has the same interpretation whether the entire matrix of observed and imputed genotypes is centered or not. In neither case does it represent the mean breeding value of selected individuals.

Accuracy of PBLUP increased with heritability, as expected (Table 3). Further, genomic predictions using panels that include causal mutations were near perfect when $\mu_{g}$was included in the model. These high accuracies are a reflection of these phenotypes being influenced by only 50 QTL and there being a large training dataset. Accuracy was reduced when $\mu_{g}$was not fitted in the model. There was no advantage in terms of accuracy to centering the covariates, but MCMC mixing may have been improved, although this was not investigated.

**Table 3:** Correlations (%, $\pm SE_{s}$) between TBV and (G)EBV for alternative analyses for different heritabilities

Heritabilities*^a^*	Analyses*^b^*				
	JC	J	C	N	PBLUP
$h^{2}=0.1$	93.68 ± 0.01	93.71 ± 0.01	92.42 ± 0.01	92.42 ± 0.01	31.33 ± 0.02
$h^{2}=0.3$	96.79 ± 0.01	96.81 ± 0.00	95.19 ± 0.01	95.19 ± 0.01	37.07 ± 0.02
$h^{2}=0.5$	97.59 ± 0.00	97.63 ± 0.00	96.32 ± 0.00	96.31 ± 0.00	41.56 ± 0.01

a Average correlation between true breeding value (TBV) and (genomic) estimated breeding values from 10 replications validated in Generation 5, comprising 8000 individuals with genotypes but no phenotypes. The true quantitative trait loci (QTL) effects were sampled from a normal distribution with mean $\mu_{\alpha }$= 0.2 and scaled to simulate a trait with heritabilities 0.1, 0.3 or 0.5.

b J: includes a covariate for *μ_g_* in the model, C: entire matrix of imputed and observed genotype covariates centered, JC: both J and C, N: neither J or C, and PBLUP: pedigree-based BLUP. Covariates were fitted for both 50 QTL and 150 markers.

**Table 4:** Regression coefficients ($\pm SE_{s}$) of TBV on (G)EBV

Genotype Data*^a^*	Analyses*^b^*				
	JC*^c^*	J	C	N	PBLUP
50 QTL + 150 markers	1.06 ± 0.02	1.06 ± 0.02	1.06 ± 0.02	1.06 ± 0.02	—
50 QTL only	1.05 ± 0.02	1.05 ± 0.02	1.12 ± 0.04	1.12 ± 0.04	—
150 markers only	0.95 ± 0.03	0.95 ± 0.03	0.95 ± 0.03	0.95 ± 0.03	—
No genotypes	—	—	—	—	0.95 ± 0.04

a Average Regression coefficients of true breeding value (TBV) on (genomic) estimated breeding values from 10 replications validated in Generation 5, comprising 8,000 individuals with genotypes but no phenotypes. The true QTL effects were sampled from a Normal distribution with mean *μ_α_*= 0.2 and scaled to simulate a trait with a trait with a heritability 0.5.

b The analyses were based on fitting covariates for only 50 QTL, only 150 markers, or both 50 QTL and 150 markers.

c J: includes a covariate for *μ_g_* in the model, C: entire matrix of imputed and observed genotype covariates centered, JC: both J and C, N: neither J or C, and PBLUP: pedigree-based BLUP.

#### Bias:

Table 4 shows the regression coefficients of TBV on (G)EBV for $h^{2}=0.5$ and $\mu_{\alpha }$ = 0.2, the same scenarios represented in Table 2. The regression coefficients of TBV on GEBV for each scenario were close to 1 with very low SE, which indicates that the genomic predictions exhibited almost no bias. The differences in regression coefficients between analyses were very small, but the marker panel comprising only markers was biased upward, whereas the marker panels that included causal mutations were biased slightly downward.

### Effect of mean QTL effect ($\mu_{\alpha }=0$ *vs.* $\mu_{\alpha }=0.2$)

We had hypothesized that the impact of omitting $\mu_{g}$ from the model would be greatest when $\mu_{g}$ departs significantly from 0, which is more likely to occur when $\mu_{\alpha }$departs from 0. For that reason, our base simulation used $\mu_{\alpha }$= 0.2. Results are shown in Table 5 for the panel including QTL and markers with $h^{2}$ = 0.5 for $\mu_{\alpha }$ = 0.2 compared with $\mu_{\alpha }$ = 0. These results confirmed that the benefit of fitting $\mu_{g}$was greatest when $\mu_{\alpha }$ = 0.2, but there was still an advantage to fitting $\mu_{g}$when $\mu_{\alpha }$= 0. That advantage is likely to erode as the number of QTL increases. Changing the mean QTL effect had no impact on bias, except for a slight influence on PBLUP.

**Table 5:** Accuracy and bias of genomic prediction ($\pm SE_{s}$) for alternative QTL distributions and analyses

Substitution*^a^* Effects	Analyses*^b^*				
	JC*^c^*	J*^d^*	C	N	PBLUP
Correlations (%)					
$\mu_{\alpha }=0$	97.91 ± 0.00	97.91 ± 0.00	97.75 ± 0.00	97.48 ± 0.00	42.66 ± 0.01
$\mu_{\alpha }=0.2$	97.59 ± 0.00	97.63 ± 0.00	96.32 ± 0.00	96.31 ± 0.00	41.56 ± 0.01
Regression coefficient					
$\mu_{\alpha }=0$	1.05 ± 0.04	1.05 ± 0.04	1.05 ± 0.04	1.05 ± 0.04	0.97 ± 0.07
$\mu_{\alpha }=0.2$	1.06 ± 0.02	1.06 ± 0.02	1.06 ± 0.02	1.06 ± 0.02	0.95 ± 0.04

a Accuracy was quantified using the average correlation between true breeding value and (genomic) estimated breeding values from 10 replications validated in Generation 5, comprising 8000 individuals with genotypes but no phenotypes.

b Bias was quantified using the average regression coefficients of true breeding value on (genomic) estimated breeding values from 10 replications.

c The true QTL effects were sampled from normal distributions with mean $\mu_{\alpha }=0$ or $\mu_{\alpha }=0.2$ and scaled to simulate a trait with a heritability 0.5.

d J: includes a covariate for $\mu_{g}$ in the model, C: entire matrix of imputed and observed genotype covariates centered, JC: both J and C, N: neither J or C, and PBLUP: pedigree-based BLUP. Covariates were fitted for both 50 QTL and 150 markers.

### Effect of more QTL and markers (200 SNP *vs.* 2000 SNP)

We had hypothesized that the improvement of accuracy from adding an extra covariate for $\mu_{g}$ would reduce as the number of QTL increases, because $\mu_{g}$is likely to be closer to zero for a trait that is more polygenic. Table 6 shows that PBLUP was largely unaffected by changes to genetic architecture, but the accuracy of genomic prediction declined slightly as the number of QTL increased. This reflects the fact that the precision of estimating QTL effects is greater when the effects are large, and polygenic traits with more QTL must have on average smaller effects when compared at the same genetic variance. The benefit of fitting $\mu_{g}$in the model was virtually eliminated when the number of substitution effects to estimate increased from 150 to 1500. In contrast to the results for accuracy, there was no impact of QTL number on bias. Centering had no impact on accuracy or bias.

**Table 6:** Accuracy and bias of genomic prediction ($\pm SE_{s}$) for different numbers of QTL and alternative analyses

Numbers of QTL*^a^*	Analyses*^b^*				
	JC*^c^*	J*^d^*	C	N	PBLUP
Correlations (%)					
50 QTL + 150 markers	97.59 ± 0.00	97.63 ± 0.00	96.32 ± 0.00	96.31 ± 0.00	41.56 ± 0.01
500 QTL + 1500 markers	90.45 ± 0.01	90.49 ± 0.01	89.99 ± 0.01	89.99 ± 0.01	41.62 ± 0.02
Regression coefficient					
50 QTL + 150 markers	1.06 ± 0.02	1.06 ± 0.02	1.06 ± 0.02	1.06 ± 0.02	0.95 ± 0.04
500 QTL + 1500 markers	1.08 ± 0.03	1.08 ± 0.03	1.08 ± 0.03	1.08 ± 0.03	0.98 ± 0.05

a Accuracy was quantified using the average correlation between true breeding value and (genomic) estimated breeding values from 10 replications validated in Generation 5, comprising 8,000 individuals with genotypes but no phenotypes.

b Bias was quantified using the average regression coefficients of true breeding value on (genomic) estimated breeding values from 10 replications.

c The true effects for 50 or 500 QTL were sampled from a Normal distribution with mean *μ_α_* = 0.2 and scaled to simulate a trait with a heritability 0.5.

d J: includes a covariate for *μ_g_* in the model, C: entire matrix of imputed and observed genotype covariates centered, JC: both J and C, N: neither J or C, and PBLUP: pedigree-based BLUP. Covariates were fitted for either 50 QTL and 150 markers or 500 QTL and 1500 markers.

### Centering using the entire matrix of genotype covariates or only the observed genotype covariates

Table 7 shows the accuracies and regression coefficients of TBV on (G)EBV for the genotype panel with 150 markers, $h^{2}=0.5$, and $\mu_{\alpha }$ = 0. The analyses were performed after centering: the entire matrix of imputed and observed genotype covariates ($M^{*}=M-\left(1/\left(n_{g}+n_{n}\right)\right)11^{\prime }M$); only observed genotype covariates ($M_{g}^{\text{*}}=M_{g}-\left(1/n_{g}\right)11^{\prime }M_{g}$), which is the type of centering done in single-step GBLUP; or not centering the covariates ($M^{*}=M$). The accuracy of the genomic analysis with covariates centered as $M_{g}^{\text{*}}$ but without **J** (model C*) was ∼17% lower than the other genomic analyses. However, when **J** was included in the model with covariates centered as $M_{g}^{\text{*}},$ the accuracy of prediction was markedly improved.

**Table 7:** Accuracy and bias of genomic prediction ($\pm SE_{s}$) when centering for all genotypes or observed genotypes

Analyses*^a^*	Correlations (%)*^b^*	Regression Coefficient*^c^*
JC	82.08 ± 0.06	0.95 ± 0.07
J	82.08 ± 0.06	0.95 ± 0.07
C	82.05 ± 0.06	0.95 ± 0.07
N	82.05 ± 0.06	0.95 ± 0.07
JC*	82.09 ± 0.06	0.95 ± 0.07
C*	65.35 ± 0.06	1.16 ± 0.11
PBLUP	42.66 ± 0.01	0.97 ± 0.07

a Accuracy was quantified using the average correlation between true breeding value and (genomic) estimated breeding values from 10 replications validated in Generation 5, comprising 8,000 individuals with genotypes but no phenotypes. The true QTL effects were sampled from Normal distributions with mean *μ_α_* = 0 and scaled to simulate a trait with a heritability 0.5.

b Bias was quantified using the average regression coefficients of true breeding value on (genomic) estimated breeding values from 10 replications.

c J: includes a covariate for *μ_g_* in the model, C: entire matrix of imputed and observed genotype covariates centered, JC: both J and C, N: neither J or C, C*: only observed genotype covariates centered, JC*: both J and C*, and PBLUP: pedigree-based BLUP. Covariates were fitted for 150 markers.

As explained previously, $\mu_{g}=k^{\prime }\alpha ,$ where $k^{\prime }$ is the expected value of the covariates in the founders, will tend to zero for the marker panel that does not include QTL, even with $\mu_{\alpha }\neq 0.$ However, even if $\mu_{\alpha }=0,$ in a population undergoing selection when selected individuals are genotyped, $\mu_{g}^{\text{*}}=\mu_{g}-v^{\prime }\alpha \neq 0,$ where $v^{\prime }$ is the expected value of the observed genotype covariates. In this study, selection was used to increase the mean of the trait. Thus, $\mu_{g}^{\text{*}}$ is expected to be negative because most of the genotyped individuals were from G5, whereas $\mu_{g}$ is expected to be zero. The negative estimate of ${\hat{\mu }}_{g}^{\text{*}}$ from 10 replicates of the JC* analysis, −2.69, confirms that $\mu_{g}^{\text{*}}<0.$ On the other hand, the mean of ${\hat{\mu }}_{g}$ from 10 replicates of the JC analysis was −0.75. This explains why fitting **J** in the model improved the accuracy of genomic prediction when covariates were centered as in single-step GBLUP.

Fernando *et al.* (2014) found that centering using $M_{g}^{\text{*}}$ improved the accuracy without **J** in the model when the population was not under selection and the genotyped individuals were unselected. In that study, mating was random with no selection, so the allele frequency means of the genotyped individuals were a reasonable approximation of the allele frequency means in the founder population. By contrast, our simulation here shows that centering using $M_{g}^{\text{*}}$ can reduce the accuracy when the population is under selection, unless **J** is fitted in the model.

In single-step GBLUP, the observed genotypes are commonly centered by subtracting their mean and used to construct a genomic relationship matrix, for example, by using the first method proposed by VanRaden (2008). Using that genomic relationship matrix in the single-step GBLUP formula in Aguilar *et al.* (2010) does not account for **J**. This was recognized by Vitezica *et al.* (2011), who proposed a modification for populations under selection that involved adding a constant to all elements of the genomic relationship matrix that they derived by equating the sum of the elements of the genomic relationship matrix to the sum of the elements of the numerator relationship matrix. In the appendix of that paper they showed that this modification is equivalent to fitting a covariate **Q** = −**J** and treating −$\mu_{g}^{\text{*}}$ as a random effect. In addition to this modification, Christensen *et al.* (2012) proposed a multiplicative scaling to the genomic relationship matrix such that its diagonals have the same mean as the diagonals of the numerator relationship matrix. Vitezica *et al.* (2011) claimed that −$\mu_{g}^{\text{*}}$ represents the mean breeding value of selected individuals, and we have confirmed here that this is true provided the observed genotype covariates are centered by their mean.

Most populations are under natural or artificial selection. In many cases, genotypes are only available on selected individuals. In single-step genomic analysis that combine genotyped and nongenotyped individuals in a joint analysis, the mean of observed genotypes are available for centering. If the observed genotypes include QTL, the accuracy of genomic prediction can be severely compromised, unless the **J** covariate is fitted in the model. If the observed genotypes are only markers, the accuracy of genomic prediction may not necessarily be improved by fitting **J** in the model, but it doesn’t do any harm. However, if centering is applied only to the observed genotypes, which is the type of centering used in single-step GBLUP, accuracy could be severely compromised, unless the **J** covariate is fitted in the model or an equivalent approach is adopted.

## Appendix

Here we show that inference about α does not depend on how the genotypes are coded. The marker effects model can be described by the following general model:

y=1μ+Mα+e, (8)

where **y** is a vector of observed phenotypes, **1** is a vector of 1s, *μ* is a general mean, **M** is a matrix of marker covariates, coded 0, 1, 2, which might represent the number of copies of the A allele, α is a vector of random substitution effects of markers, and **e** is a vector of residuals. Henderson’s mixed model equations (MME) that correspond to Equation 8 are:

$$\left[\begin{array}{cc} 1^{\prime }1 & 1^{\prime }M\\ M^{\prime }1 & M^{\prime }M+I\frac{\sigma_{e}^{2}}{\sigma_{\alpha }^{2}} \end{array}\right]\left[\begin{array}{c} \hat{\mu }\\ \hat{\alpha } \end{array}\right]=\left[\begin{array}{c} 1^{\prime }y\\ M^{\prime }y \end{array}\right],$$

where $\hat{\mu }$ is the best linear unbiased estimate of the mean, and $\hat{\alpha }$ is the best linear unbiased predictor of the vector of random substitution effects of all markers. Now we can eliminate $\hat{\mu }$ from the equations for $\hat{\alpha },$ by subtracting from those equations the equation for $\hat{\mu }$ premultiplied by $M^{\prime }1/n.$ Then, the MME are transformed to

$$\left[\begin{array}{cc} 1^{\prime }1 & 1^{\prime }M\\ 0 & M^{\prime }M-\frac{M^{\prime }11^{\prime }M}{n}+I\frac{\sigma_{e}^{2}}{\sigma_{\alpha }^{2}} \end{array}\right]\left[\begin{array}{c} \hat{\mu }\\ \hat{\alpha } \end{array}\right]=\left[\begin{array}{c} 1^{\prime }y\\ M^{\prime }y-\frac{M^{\prime }1}{n}1^{\prime }y \end{array}\right],$$

and substituting $1^{\prime }1=n$, and $1^{\prime }M=n\bar{m^{\prime }\prime }$ and its transpose, the transformed MME become

$$\left[\begin{array}{cc} n & n\bar{m}\\ 0 & M^{\prime }M-\bar{m}{\bar{m}}_{\prime }n+I\frac{\sigma_{e}^{2}}{\sigma_{\alpha }^{2}} \end{array}\right]\left[\begin{array}{c} \hat{\mu }\\ \hat{\alpha } \end{array}\right]=\left[\begin{array}{c} 1^{\prime }y\\ {\left(M-1{\bar{m}}^{\prime }\right)}^{\prime }y \end{array}\right].$$ (9)

where ${\bar{m}}^{\prime }\prime$ is the row vector of column means of M as in ${\bar{m}}^{\prime }\prime =1^{\prime }M/n.$

Now, consider the coding obtained by centering the marker genotypes as $M-1{\bar{m}}^{\prime }\prime .$ Then the model can be written as

$$y=1\mu^{*}+\left(M-1{\bar{m}}^{\prime }\right)\alpha +e,$$ (10)

where $\mu^{*}=\mu +{\bar{m}}^{\prime }\alpha .$ The MME that correspond to Equation 10 are

$$\left[\begin{array}{cc} n & 1\prime \left(M-1{\bar{m}}^{\prime }\prime \right)\\ {\left(M-1{\bar{m}}^{\prime }\prime \right)}^{\prime }1 & {\left(M-1{\bar{m}}^{\prime }\prime \right)}^{\prime }\left(M-1{\bar{m}}^{\prime }\prime \right)+I\frac{\sigma_{e}^{2}}{\sigma_{\alpha }^{2}} \end{array}\right]\left[\begin{array}{c} {\hat{\mu }}^{\text{*}}\\ \hat{\alpha } \end{array}\right]=\left[\begin{array}{c} 1^{\prime }y\\ {\left(M-1{\bar{m}}^{\prime }\prime \right)}^{\prime }y \end{array}\right],$$

but $1^{\prime }\left(M-1{\bar{m}}^{\prime }\prime \right)=n{\bar{m}}^{\prime }\prime -n{\bar{m}}^{\prime }\prime =0^{\prime },$ and, similarly, its transpose is ${\left(M-1{\bar{m}}^{\prime }\prime \right)}^{\prime }1=0.$ Then the MME become

$$\left[\begin{array}{cc} n & 0^{\prime }\\ 0 & {\left(M-1{\bar{m}}^{\prime }\prime \right)}^{\prime }\left(M-1{\bar{m}}^{\prime }\prime \right)+I\frac{\sigma_{e}^{2}}{\sigma_{\alpha }^{2}} \end{array}\right]\left[\begin{array}{c} {\hat{\mu }}^{\text{*}}\\ \hat{\alpha } \end{array}\right]=\left[\begin{array}{c} 1^{\prime }y\\ {\left(M-1{\bar{m}}^{\prime }\prime \right)}^{\prime }y \end{array}\right].$$

Expanding ${\left(M-1{\bar{m}}^{\prime }\prime \right)}^{\prime }\left(M-1{\bar{m}}^{\prime }\prime \right)$ gives $M^{\prime }M-\bar{m}\prime 1^{\prime }M-M^{\prime }1{\bar{m}}^{\prime }\prime +\bar{m}1^{\prime }1{\bar{m}}^{\prime }\prime ,$ but because $1^{\prime }M=n{\bar{m}}^{\prime }\prime ,$ $M^{\prime }1=n\bar{m}\prime ,$ and $1^{\prime }1=n,$ as previously shown, the second term, $\bar{m}1^{\prime }M=\bar{m}n{\bar{m}}^{\prime }\prime ,$ which is equal to the last term in the expansion. Thus, the MME become

$$\left[\begin{array}{cc} n & 0^{\prime }\\ 0 & M^{\prime }M-\bar{m}{\bar{m}}^{\prime }n+I\frac{\sigma_{e}^{2}}{\sigma_{\alpha }^{2}} \end{array}\right]\left[\begin{array}{c} {\hat{\mu }}^{\text{*}}\\ \hat{\alpha } \end{array}\right]=\left[\begin{array}{c} 1^{\prime }y\\ {\left(M-1{\bar{m}}^{\prime }\prime \right)}^{\prime }y \end{array}\right].$$ (11)

The equations for $\hat{\alpha }$ in (9) and (11) are identical, and this proves that centering with $m^{\prime }$ doesn’t affect inference about α.

Now suppose an arbitrary vector $v^{\prime }$ is used to transform the genotypes as $\left(M-1v^{\prime }\right).$ Then the model becomes

$$y=1\left(\mu +v^{\prime }\alpha \right)+\left(M-1v^{\prime }\right)\alpha +e.$$

Adding and subtracting $1{\bar{m}}^{\prime }\prime \alpha ,$ the above equation can be written a:

$$\begin{array}{c} y=1\left[\mu +v^{\prime }\alpha +\left({\bar{m}}^{\prime }-v^{\prime }\right)\alpha \right]+\left(M-1{\bar{m}}^{\prime }\prime \right)\alpha +e\\ =1\left(\mu +{\bar{m}}^{\prime }\prime \alpha \right)+\left(M-1{\bar{m}}^{\prime }\prime \right)\alpha +e\\ =1\mu^{*}+\left(M-1{\bar{m}}^{\prime }\prime \right)\alpha +e, \end{array}$$

which is identical to Equation 10, proving that inference about α does not depend on how the genotypes are coded.
